# Genome-wide association study in Chinese cohort identifies one novel hypospadias risk associated locus at 12q13.13

**DOI:** 10.1186/s12920-019-0642-0

**Published:** 2019-12-19

**Authors:** Zhongzhong Chen, Xiaoling Lin, Yunping Lei, Haitao Chen, Richard H. Finnell, Yaping Wang, Jianfeng Xu, Daru Lu, Hua Xie, Fang Chen

**Affiliations:** 1 Department of Urology, Shanghai Children’s Hospital, Shanghai Jiao Tong University, Shanghai, 200062 China; 2 State Key Laboratory of Genetic Engineering, Collaborative Innovation Center for Genetics and Development, School of Life Sciences, Fudan University, Shanghai, 200438 China; 3 Department of Urology, Huashan Hospital, Fudan University, Shanghai, 200040 China; 4 Center for Precision Environmental Health, Departments of Molecular and Cellular Biology and Medicine, Baylor College of Medicine, Houston, TX 77030 USA; 5 Department of Urology, Shanghai Jiao Tong University Affiliated Sixth People’s Hospital, Shanghai, 200233 China; 6 Shanghai Eastern Urological Reconstruction and Repair institute, Shanghai, 200233 China

**Keywords:** Hypospadias, Genome-wide association study, SP1, 12q13.13, Chinese

## Abstract

**Background:**

Hypospadias risk–associated gene variants have been reported in populations of European descent using genome-wide association studies (GWASs). There is little known at present about any possible hypospadias risk associations in Han Chinese populations.

**Methods:**

To systematically investigate hypospadias risk–associated gene variants in Chinese patients, we performed the first GWAS in a Han Chinese cohort consisting of 197 moderate-severe hypospadias cases and 933 unaffected controls. Suggestive loci (*p* < 1 × 10^− 4^) were replicated in 118 cases and 383 controls, as well as in a second independent validation population of 137 cases and 190 controls. Regulatory and protein-protein interactions (PPIs) were then conducted for the functional analyses of candidate variants.

**Results:**

We identified rs11170516 with the risk allele G within the *SP1*/*SP7* region that was independently associated with moderate-severe hypospadias [*SP1*/*SP7*, rs11170516, *P*_combine_ = 3.5 × 10^− 9^, odds ratio (OR) = 1.96 (1.59–2.44)]. Results also suggested that rs11170516 is associated with the expression of *SP1* as a cis-expression quantitative trait locus (cis-eQTL). Protein SP1 could affect the risk of hypospadias via PPIs.

**Conclusions:**

We performed the first GWAS of moderate-severe hypospadias in a Han Chinese cohort, and identified one novel susceptibility *cis*-acting regulatory locus at 12q13.13, which may regulate a variety of hypospadias-related pathways by affecting proximal *SP1* gene expression and subsequent PPIs. This study complements known common hypospadias risk-associated variants and provides the possible role of *cis*-acting regulatory variant in causing hypospadias.

## Background

Hypospadias, arising during weeks of 8–16 of gestation [1], is among the most common congenital diseases of the male uro-genital system. Hypospadias occurs in approximately 1 per 1000 births in China [2], and the prevalence is suggested to be increasing here [3]. In addition, the incidence seems to vary by urban-rural classification and geographical location [2]. Hypospadias severity is classified into mild (in glandular), moderate (in penile) and severe (in scrotum or perineum) according to the abnormal location of the urethral opening [4–6]. Many hypospadias patients have concurrent complications, and children with hypospadias repaired in childhood usually reemerge in adulthood that making the patient feel traumatized [7].

Hypospadias is considered to be a complex congenital disorder stemming from multiple genetic and environmental interacting factors [8]. Investigation of the familial aggregation of hypospadias cases suggested that genetic, rather than intrauterine environmental factors, play a principal role in the etiology of hypospadias [9]. The heritability of hypospadias is between 57 and 77% [9, 10]. Hypotheses about the multifactorial (polygenic) inheritance of hypospadias indicated that small effects of multiple genes and/or environmental factors might influence an individual’s hypospadias risk. The current literatures is replete with a number of genes and pathways that are known to contribute to the etiology of hypospadias, including: HH (Hedgehog) signaling pathway, WNT signaling pathway, FGF signaling pathway, BMP signaling pathway, Homeobox genes and others [11]. Associated polymorphisms with hypospadias were found in *DGKK*, *SRD5A2*, *ESR1*, *ESR2*, *FGF8*, *FGFR2*, *HSD17B3*, *MID1*, *CYP1A1*, *ATF3*, *MAMLD1*, *GSTM1*, *GSTT1* and *AR* [12]. Despite the large number of genes contributing to the etiology of hypospadias, the majority of genetic risk factors remain largely unknown. A genome-wide association study (GWAS) is an effective way to identify genetic variants associated with different human disorders while providing valuable insights into their genomic architecture. To date, GWASs for hypospadias have identified approximately 24 susceptibility loci, capable of explaining no more than 9.4% of the variance in liability to hypospadias [13, 14]. Recently, SNPs in *HAAO* and *IRX6* genes were found to be associated with hypospadias in Japanese population [15]. However, these GWASs or association analysis were performed in individuals of European descent or Japanese population, no GWAS analysis of hypospadias has yet been conducted in Chinese populations.

In the present study, we performed a GWAS based on 197 moderate-severe hypospadias cases and 933 controls in a cohort of male Han Chinese using the Illumina Omni chips, followed by two additional independent confirmation studies of males at differing life stages that included 255 cases and 573 controls. Regulatory and protein-protein interactions (PPIs) were then conducted for the functional analysis of candidate non-coding variants.

## Methods

### Study cohorts and design

We conducted a three-stage case–control study design in a male Han Chinese population. The three stages consisted of a discovery and two replication stages (Additional file 1: Table S1). Subjects of the discovery GWAS stage included 200 hypospadias cases and 1008 healthy controls, followed by replication in two independent sample sets. Subjects for the first replication stage included 118 cases and 383 controls. Subjects for the second replication stage were comprised of 137 cases and 190 controls. And the subjects in the discovery GWAS stage, replication 1 and replication 2 were recruited in three different periods. Cases were pathologically diagnosed hypospadias recruited from the Department of Urology at Shanghai Children’s Hospital. According to the abnormal location of the urethral opening, the patients were divided into three categories: mild (glandular), moderate (penile), or severe (in the scrotum or perineum). Only patients with moderate-severe hypospadias without other system abnormalities were included in our analysis. Of the 455 patients ultimately enrolled (3.2 ± 2.7 years), there were 34.9% moderate cases and 65.1% severe cases. Among the 1581 healthy controls, 1008 healthy controls (62.1 ± 10 years) were cancer-free at the time of enrollment and were recruited from the Chinese Consortium for Prostate Cancer Genetics (ChinaPCa) [16, 17] and 573 hypospadias-free controls (5.7 ± 3.3 years) were collected from subjects receiving routine physical examination in Shanghai Children’s Hospital. All these 573 healthy controls were confirmed to have a normal position of the external urethral orifice, noncleaved prepuce and intrascrotal testis.

Protocols were reviewed and approved by the Ethics Committee of the Shanghai Children’s Hospital in China (2014R022-F01). All of the samples were obtained from all participants or their parent/legal guardian with the written informed consent.

### Genotyping and quality control of discovery GWAS

In the discovery phase study, genotyping was conducted using the Illumina Human OmniExpress BeadChips. A total of 887,270 SNPs were genotyped in 200 cases and 1008 controls. A series of quality control (QC) filtering steps were applied to select samples and SNPs for further analyses. We removed samples according to four QC criteria: (1) overall missing genotype data that exceed > 5%; (2) discordant sex information; (3) duplicated or questionable familial relationships [Identity-by-state (IBS) similarity score > 0.99]; (4) individuals with scores at least six standard deviations of principal components from the sample mean score. After sample quality control analysis, a total of 197 cases and 933 controls with 530,907 SNPs remained. Genotypes in the GWAS were imputed for ~ 3.5 million SNPs using the 1000 Genomes Project Han Chinese in Beijing (CHB) population as a reference.

SNPs were excluded using the following 4 QC criteria: (1) average call rate in cases and controls and overall call rate < 95%; (2) MAF (minor allele frequency) in controls is < 5%; (3) Hardy-Weinberg Equilibrium (HWE) *p*-value in controls is < 0.001; (4) SNPs with ambiguous calls (A/T or C/G). After SNP quality control analysis, ~ 3.0 million SNPs were available for further analysis.

### SNP selection and genotyping in replication studies

We attempted to follow the general steps for selecting susceptibility SNPs identified in the GWAS discovery stage for the further confirmation (replication 1 and replication 2). We selected a subset of independently hypospadias risk-associated SNPs for replication on the basis of the following three criteria: (i) *P* < 1 × 10^− 4^ in the association test (370 SNPs met this criterion), (ii) linkage disequilibrium (LD) r^2^ below 0.5 between markers (62 SNPs met both of these criteria) and (iii) SNPs with an allele frequency difference of ≤0.02 between control subjects from the GWAS stage and subjects from 1000 Genomes Project CHB population (22 SNPs met all three criteria). Finally, genotyping of the 22 significantly associated SNPs (Additional file 2: Table S2) in the initial GWAS stage was conducted using the MassARRAY iPLEX (Sequenom) in independent replication 1 (118 cases and 383 controls) and 2 (137 cases and 190 controls). Among these 22 SNPs, three SNPs [rs6685335 (Chr 1), rs34709644 (Chr 4) and rs7805909 (Chr 7)] failed genotyping in over 5% of the samples. Therefore, a total of 19 SNPs were genotyped in replication 1 and 2 (Additional file 3: Table S3). In the current study, the associations of previous reported SNPs identified in European GWAS [13] were evaluated. We also tested associations of identified SNP separately for the moderate and the severe hypospadias patients.

### Regulatory and protein-protein network analysis

The correlations between the candidate SNP genotype and gene expression and protein binding were examined using data available from the RegulomeDB database [18] and UCSC Genome Browser database [19]. We also investigated the impact of non-coding variants using HaploReg that integrated LD information [20]. LD structure in the vicinity of the risk loci was also inferred by the Ensembl database [21]. The protein-protein network visualization was further used to better understand the biological processes mediated by the hypospadias associated risk genes based on STRING [22] and GeneSense [23]. Only those PPI pairs with a STRING combined score ≥ 700 were selected for the network analysis.

### Statistical analyses

Logistic regression analyses under log-additive model were applied to test for associations between hypospadias and controls. Population stratification and sample quality control were accounted for by principal component analysis (PCA) using the PLINK software package (http://pngu.mgh.harvard.edu/~purcell/plink/) [24]. Principal component plots were conducted using the R statistical program (http://cran.r-project.org/). Cochran’s Q statistic and the I^2^ index were accomplished to assess the heterogeneity of the SNP associations across studies. Regional plots were performed using LocusZoom [25]. Statistical analyses were performed using R and PLINK [24].

## Results

### GWAS analysis

**Fig. 1:**
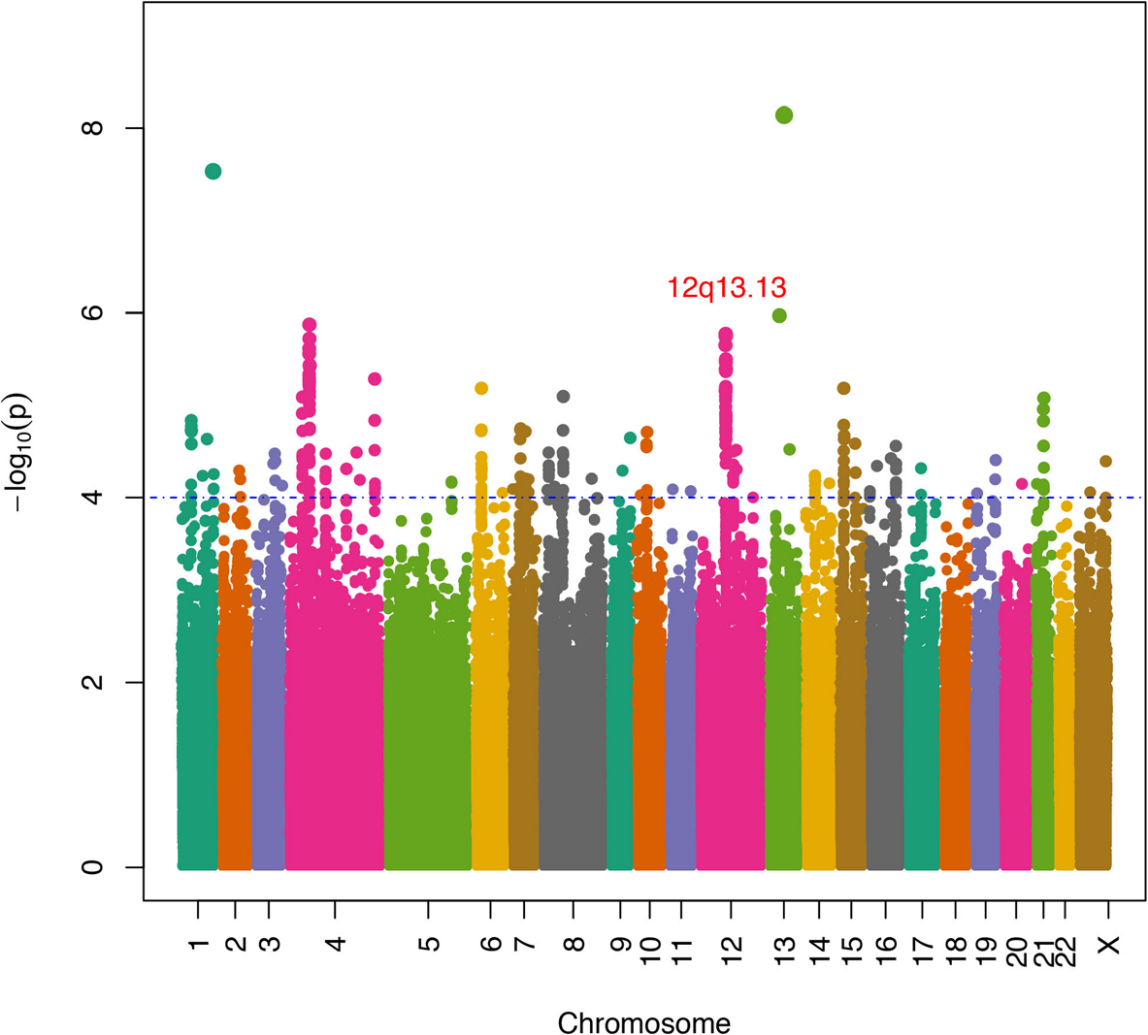
Manhattan plot of the genetic evidence of association for hypospadias in a Chinese population using log-additive model. The x-axis shows the chromosomal position, and the y-axis represents the –log10 *P*additive value. The horizontal dashed blue line indicates the preset threshold of *P* = 10^− 4^

**Fig. 2:**
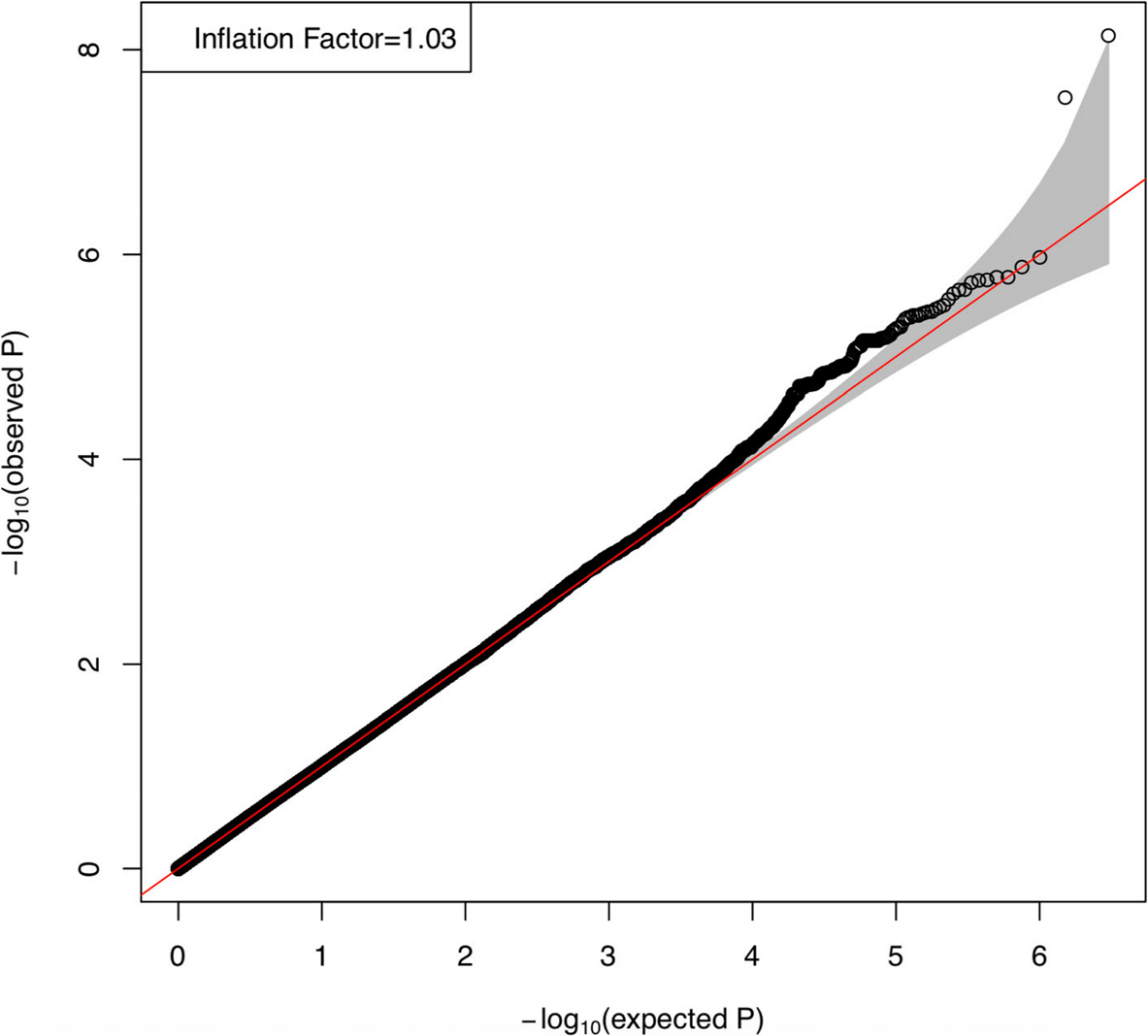
The quantile-quantile (Q-Q) plot of expected *P* values versus observed P values in hypaspadias. The red line shows the distribution under the null hypothesis while the shaded band represents 95% CI values

In the first discovery stage, 887,270 SNPs were genotyped in 200 cases and 1008 Han Chinese controls using the Illumina Human OmniExpress BeadChips. Genotypes in the GWAS were imputed using the 1000 Genomes Project Han Chinese in Beijing (CHB) population as a reference population. After quality control filtering, 3,015,028 SNPs in 197 cases and 933 controls qualified for subsequent analysis. We conducted association analysis for each SNP under a log-additive model and identified multiple hypospadias risk-associated regions (Fig. 1). Association analysis of ~ 3.0 million genetic variants with hypospadias in the discovery stage in the Han Chinese population demonstrated little evidence of global test statistic inflation (inflation factor = 1.03) caused by population stratification (Fig. 2). This result was further supported by principal component analysis (PCA) (Additional file 7: Figure S1).

We first tested for association at known GWAS loci identified by a European GWAS. Among the 18 previously described hypospadias-associated SNPs, 16 SNPs passed the quality controls. Among these 16 SNPs examined, we identified 1 SNP (rs4554617) in *DGKK* with significant association (*P* = 1.4 × 10^− 3^) through logistic regression (additive model) (Additional file 4: Table S4). To evaluate additional susceptibility genetic loci, we selected 22 SNPs representing the top associated loci for replication/validation studies (Additional file 2: Table S2). From these loci, only 1 SNP (rs11170516) with a risk allele (G allele) showed significant association at each stage [discovery stage: odds ratio (OR) = 2.27, *P* = 1.6 × 10^− 6^; replication 1: OR = 1.69, *P* = 1.3 × 10^− 2^; replication 2: OR = 1.79, *P* = 8.0 × 10^− 3^] (Table 1). After combining the results from all three stages using a meta-analysis assuming a fixed effect, association of rs11170516 at 12q13.13 exceeded genome-wide significance [OR = 1.96, 95% confidence interval (CI) = 1.59–2.44; *P* = 3.5 × 10^− 9^]. There was no evidence for heterogeneity across the three stages at rs11170516 (*P*_het_ = 0.52, I^2^ = 0). In addition, the rs11170516 is significantly associated with moderate hypospadias (OR = 1.83, *P* = 4.9 × 10^− 4^) and severe hypospadias (OR = 2.03, *P* = 1.64 × 10^− 7^) separately (Additional file 5: Table S5). In the present study, the rs11170516 risk allele frequencies (RAF) were 77.9% in control of discovery stage, 79.8% in control of replication 1, and 78.3% in control of replication 2, respectively. These frequencies are very similar to HAPMAP-CHB, which reported RAF value is 80.5%.

**Table 1:** Association results for the three stages and the meta-analysis at the 12q13.13 locus

SNP	Risk Allele	Location	Attributed genes	Study	RAF^a^		OR(95% CI)^b^	*P* value^b^	*P*het^c^
					Cases	Controls			
rs11170516	G	12:53752692	*SP1*, *SP7*	Discovery stage	0.888	0.779	2.27(1.64–3.23)	1.6 × 10^− 6^	0.52
				Replication 1	0.871	0.798	1.69(1.14–2.63)	1.3 × 10^−2^	
				Replication 2	0.865	0.783	1.79(1.16–2.63)	8.0 × 10^−3^	
				Meta-analysis	0.877	0.784	1.96(1.59–2.44)	3.5 × 10^−9^	

^a^Risk allele frequency (RAF)

^b^ORs, 95% CIs and corresponding *P* values in additive model were estimated using a logistic regression model

^c^*P* value of Cochran’s Q-test for the heterogeneity

### Functional analysis

**Fig. 3:**
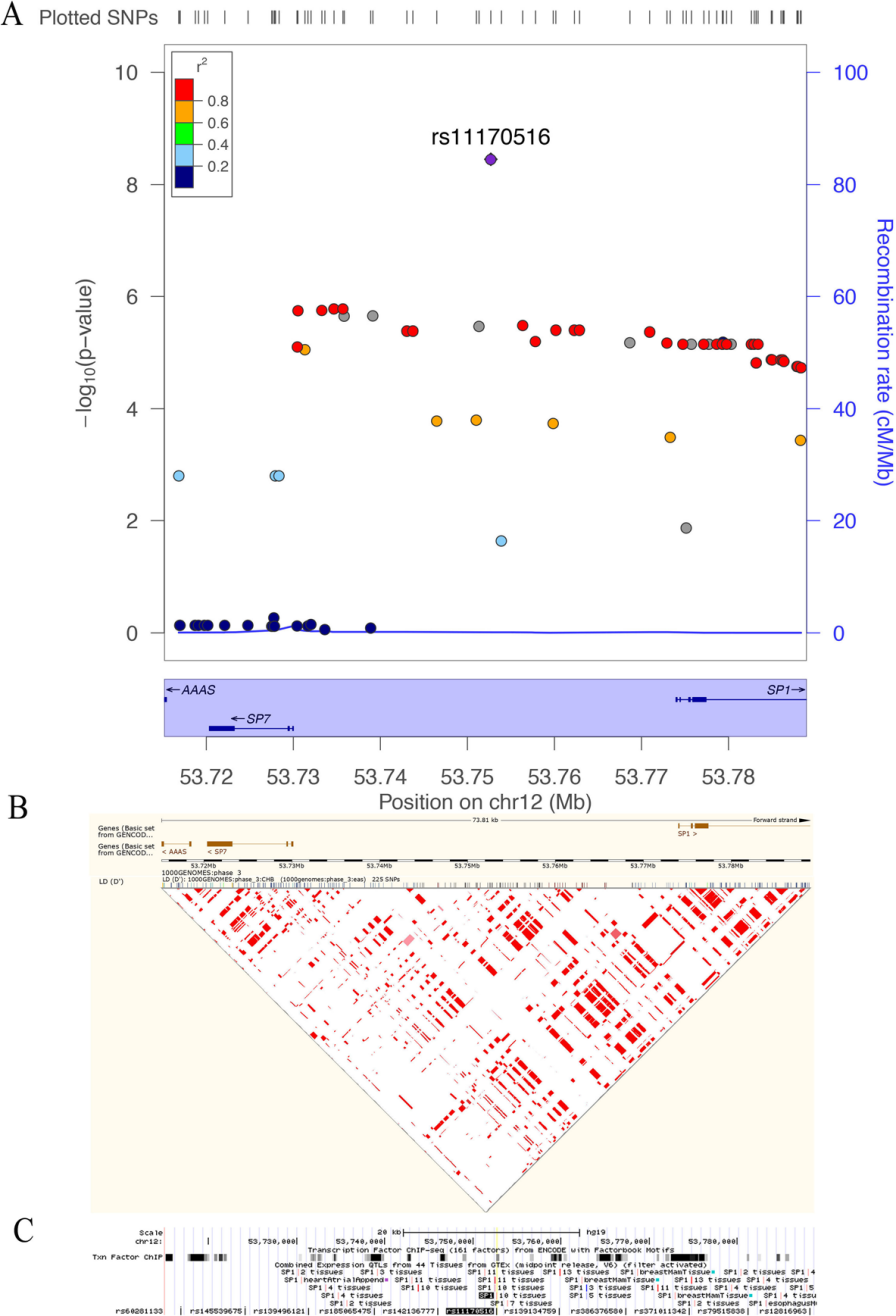
Regional association results (top), LD plots (middle and bottom) and functional annotation for new hypospadias susceptibility region at 12q13.13. (**a**) For regional plots, genomic coordinates are shown on the x axis (hg19/GRCh37), *P* values for the association analysis are plotted as –log_10_*P* against chromosomal position on the left y axis. *P* value of rs11170516 was calculated based on the combined three stage results. Both genotyped and imputed SNPs are shown. The right y axis represents the recombination rate estimated from 1000 Genomes Project ASN data. (**b**) LD heat maps based on D′ values using CHB genotypes from the 1000 Genomes Project (Phase 3) according to Ensembl annotations. (**c**) rs11170516 affected *SP1* expression by the UCSC browser

**Fig. 4:**
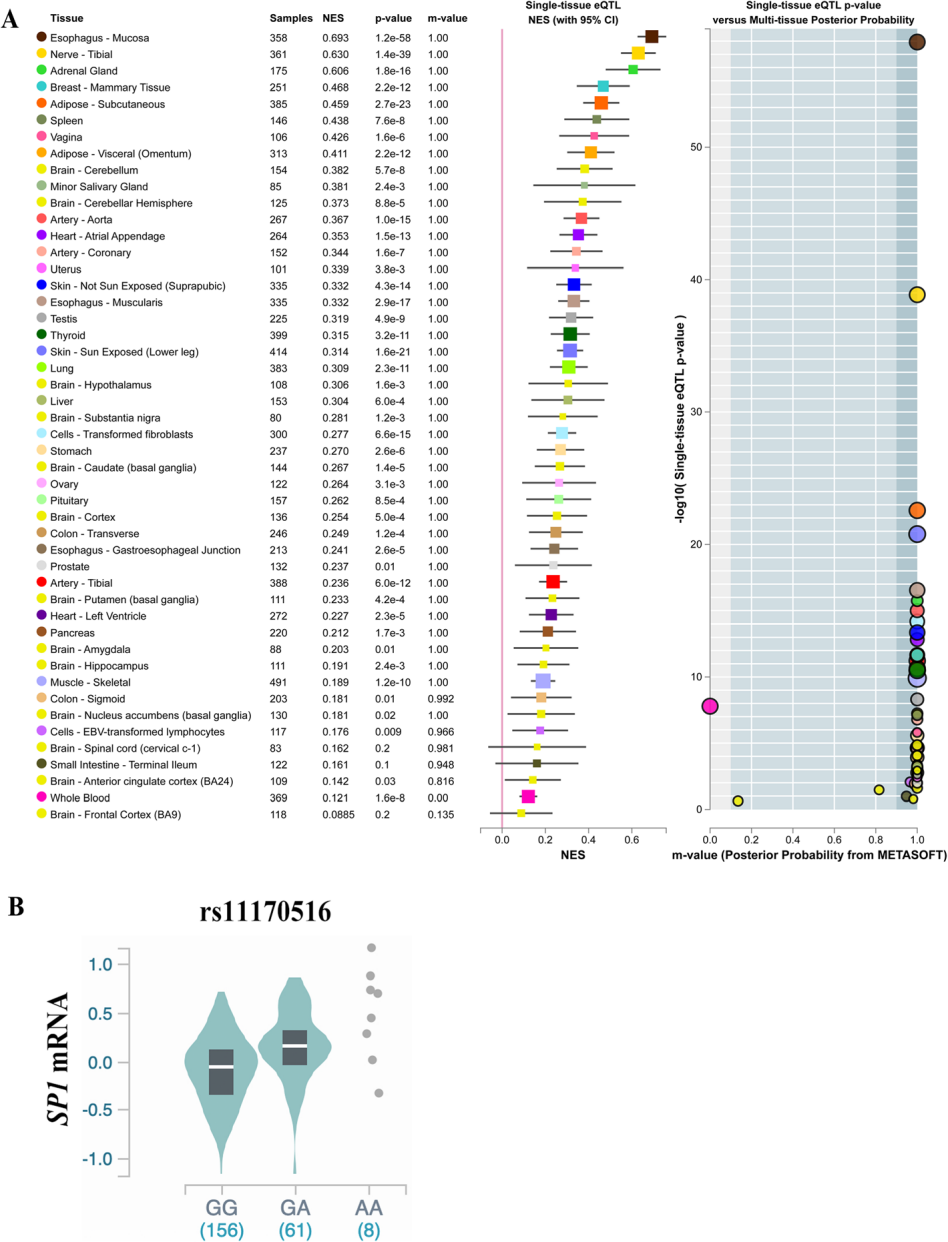
eQTL analysis for rs11170516. (**a**) Association of rs11170516 with *SP1* expression in multi tissues. (**b**) Association of rs11170516 with *SP1* expression in testis. eQTL data were obtained from GTEx portal (www.gtexportal.org)

**Fig. 5:**
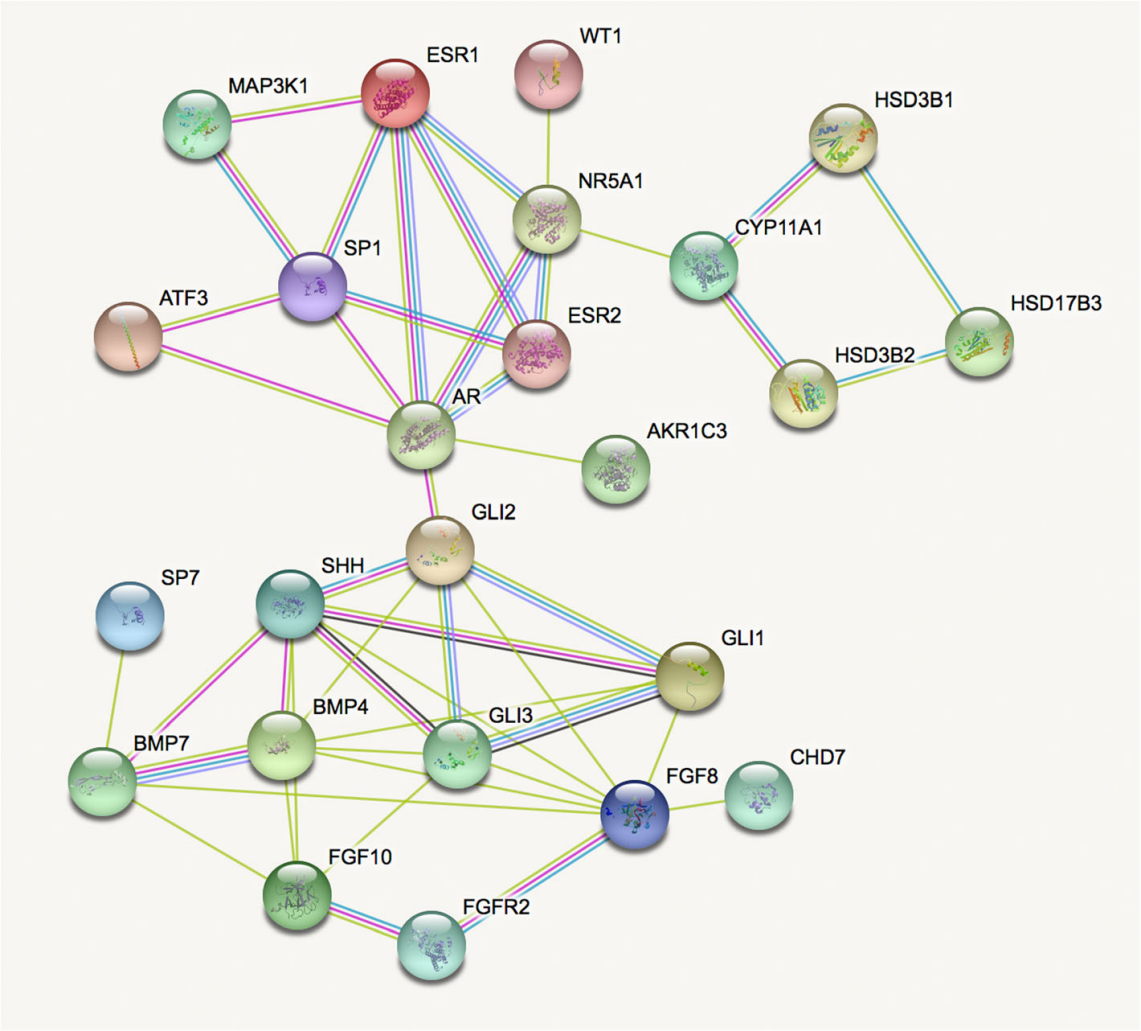
Protein-protein interactions (PPIs) network of SP1, SP7 proteins and proteins encoded by known hypospadias risk associated genes in human

The most significant SNP in this study, was determined to be rs11170516 at 12q13.13, located in the intergenic region ~ 23 kbp 5′ of the *SP7* (Sp7 transcription factor) gene and at ~ 21 kbp 5′ of the *SP1* (Sp1 transcription factor) gene (Fig. 3a). Further analysis showed that a linkage disequilibrium (LD) block containing rs11170516 overlaps with *SP1* (Fig. 3b) based on the Ensembl database [21]. Further, 22 SNPs of *SP1* were found to be in strong LD (r^2^ ≥ 0.8) with rs11170516 using HaploReg database (Additional file 6: Table S6). A bioinformatics analysis of this region, based on Genotype-Tissue Expression (GTEx) data annotated by the UCSC browser [19], revealed that the genetic variant rs11170516 is likely affecting proximal *SP1* gene expression (Fig. 3c). The variant is significantly associated with the expression of *SP1* mRNA level in multiple tissues including testis, uterus and vagina (Fig. 4a), and the risk allele of rs11170516 is associated with low expression of *SP1* (Fig. 4b). We also investigated *cis*-eQTL effects and the regulatory protein binding of rs11170516 (Table 2) using RegulomeDB annotation [18]. Again, SNP rs11170516 was identified as a *cis*-eQTL and it is associated with *SP1* expression in monocytes [27] (Table 2). Furthermore, the expression of *SP1* was relatively higher in skin based on data from the NCBI browser (Additional file 8: Figure S2). The regulatory protein binding information in the RegulomeDB annotation [18] showed that rs11170516 is bound by the regulatory protein ESR1 (estrogen receptor 1) based on the ENCODE database [26] (Table 2). To further interpret the potential mechanism regulated by *SP1* and *SP7*, we investigated these two genes and previous reported hypospadias risk associated genes [11] by PPIs analysis using GeneSense [23] and STRING [22]. Our results demonstrated that SP1 directly interacts with five proteins encoded by hypospadias risk genes, involving four signaling pathways, being AR (androgen receptor) in androgen production and signaling, ESR1 and ESR2 in estrogen production and signaling, MAP3K1 in gonadal development and signaling, and ATF3 in the estrogen pathway (Fig. 5). In total, 79% (22/28) of the proteins encoded by previous reported hypospadias risk associated genes [11] directly or indirectly interacted with SP1 and SP7, whereas six proteins (DGKK, SRD5A2, HOXA4, HOXB6, MAMLD1, BNC2) were not found to be interacted with SP1 and SP7.

**Table 2:** SNP rs11170516 is likely to affect protein binding of ESR1 and interact with gene *SP1* based on RegulomeDB annotation

Method	Location	Bound protein/affected gene	Cell type	Additional info	Reference
ChIP-seq	chr12:53752659..53752963	ESR1	ECC-1	estradiol_10nm	ENCODE^a^
eQTL	chr12:53752691..53752692	*SP1*	Monocytes	cis	20,502,693

^a^The Encyclopedia of DNA Elements [26]

## Discussion

To our knowledge, there is as yet no large-scale genome-wide analysis on potential genetic associations with hypospadias risk in a Han Chinese population. By performing a GWAS analysis with 452 moderate-severe hypospadias cases and 1581 controls, we identified a single locus at 12q13.13 that was associated with moderate-severe hypospadias (Table 1). Although the sample size at the initial GWAS discovery stage was relatively small, our three-stage GWAS provided convincing evidence that the 12q13.13 region is associated with the development of moderate-severe hypospadias. We identified the rs11170516 with risk allele G (the major allele) as the most significantly moderate-severe hypospadias associated SNP at this locus [*P*_combine_ = 3.5 × 10^− 9^, OR = 1.96 (1.59–2.44)], and we believe that this variant might affect the expression of the proximal *SP1* gene and subsequently regulate a variety of hypospadias-related pathways. The rs11170516 RAF is very similar between controls and the HAPMAP-CHB cohort.

Previously performed GWAS involving Dutch and Swedish populations proposed *DGKK* as a major risk gene for hypospadias [14], while a GWAS dataset from Denmark, Netherlands and Sweden identified several significant loci within or close to genes (*HOXA4*, *IRX5*, *IRX6* and *EYA1*) with important roles in embryonic development [13]. Among these associated genes, *HOXA4* mutations have been found in cases of hypospadias and showed a direct link to this malformation [28]. *HOXA4*, part of the A cluster on chromosome 7, is spatially and temporally regulated during embryonic development. Other *HOX* family genes, affected by regulatory mutation, also cause limb malformations [29]. Chromosome 12q13.13 deletions including the *HOXC* cluster were found to result in developmental delay and skeletal anomalies [30]. In addition, other genes in 12q13.13 region might also contribute to the genetic etiology of mild bone-related dysmorphism [31]. Interstitial microduplication 12q13.2-q13.3 were identified in a patient with hypospadias, dysmorphism, developmental delay and atypical seizures [32], indicating that the gene variants within 12q13 might play an important role in the etiology of hypospadias. However, no SNPs in the 12q13 region have yet been associated with hypospadias in a large cohort. Our GWAS dataset was the first to identify a significant association between SNP rs11170516 mapped to the 12q13.13 region and hypospadias in a Han Chinese population. Although we think that the association between the variant and hypospadias is mediated via *SP1*, it could also be another mechanism. Interestingly, the 12q13.13 region also contains *HOXC* cluster. This study provides new insights into the genetic etiology of hypospadias, as well as provided potential mechanisms underlying the development of this defect.

The genetic variant rs11170516 at 12q13.13 resides in an intergenic region between the transcription factor gene *SP1* and *SP7*. The protein encoded by the *SP1* gene is involved in multiple fundamental cellular processes, including: cell growth, differentiation, immune responses, apoptosis, response to DNA damage, and chromatin remodeling. The activity of this variant gene product could be significantly altered by many post-translational modifications such as acetylation, phosphorylation, glycosylation, and proteolytic processing (provided by RefSeq, Nov 2014). Osterix (Osx/Sp7), a member of the Sp family, is required for bone formation during embryonic development [33], as well as in growing and adults bones [34–36]. Sp7 can bind canonical Sp1 cognate elements [37] and its transcriptional activity requires the recruitment of Sp1 [33]. Strong LD between rs11170516 and a variety of loci in *SP1* (r^2^ > 0.8) was observed (Fig. 3b and Additional file 5: Table S5), as well as the *cis*-eQTL effects of rs11170516 on *SP1* expression (Fig. 3c and Table 2). Although we did not include all possible proteins encoded by hypospadias risk associated genes (for example IRX5, IRX6, EYA1) for PPIs, our investigations indicated that disruption of SP1 and SP7 activity is associated with multiple known hypospadias risk associated genes [11] in human via PPIs. Although it is interesting that 79% of the proteins encoded by hypospadias risk genes interact with SP1 or SP7, this is not very surprising: many of the hypospadias risk genes come from candidate gene studies, and the genes in these studies were often selected based on their interaction with another known hypospadias risk gene. So if SP1 or SP7 interacts with one of the proteins encoded by a hypospadias risk gene, it is likely to interact (indirectly) with many of the proteins encoded by hypospadias risk genes. This can also be seen in Fig. 5; most proteins are linked to SP1 or SP7 via other proteins. SP1 interacts directly with five proteins, including: AR, ESR1, ESR2, MAP3K1 and ATF3 (Fig. 5). AR mutations were observed in 3.3% of the isolated hypospadias cohort, and play an important role in the cause of hypospadias [38]. *ESR1* SNPs and haplotypes influence the risk of hypospadias in nonHispanic white and Hispanic population [39], while variants in *ESR2* are associated with hypospadias in the Swedish cohort [40]. Mutations of *MAP3K1* and *ATF3* were also found to be associated with an increased risk of hypospadias [41, 42]. Based upon the above data, we hypothesize that *SP1* is a candidate hypospadias associated gene. Considering that *SP1* is likely to be dosage sensitive [43], rs11170516 may affect proximal *SP1* gene expression in a minor effect, which can disturb a variety of downstream pathways including: androgen production and signaling, estrogen production and signaling, gonad development and signaling and estrogen pathway. Additional genetic and functional characterization of this SNP and other loci are needed to further delineate the mechanism by which the 12q13.13 locus contributes to the etiology and expression of hypospadias.

There are three limitations to our study. First, our sample size is not sufficiently large at the initial GWAS discovery stage to identify all of the possible genetic susceptibility loci associated with hypospadias. Second, although this is the first GWAS in a Han Chinese cohort to assess hypospadias risk, the study cohort is limited to moderate and severe hypospadias. Therefore, the current study may miss potentially important SNPs that are associated with mild hypospadias. Finally, rare variants, poorly covered by GWAS technological approaches, may contribute to the “missing heritability” in hypospadias. Ongoing efforts are underway to uncover all of the elements that contribute to the genetic etiology of hypospadias.

## Conclusions

In summary, we conducted the first GWAS of moderate-severe hypospadias in a Han Chinese cohort and identified one new susceptibility locus (rs11170516) at 12q13.13, which may regulate a variety of hypospadias-related pathways by affecting proximal *SP1* gene expression. These findings complement known common hypospadias risk-associated gene variants, and suggests the potential role of a *cis*-acting regulatory variant in causing moderate-severe hypospadias. Functional consequence of the SNP rs11170516 needs further validation.

## Supplementary information

**Additional file 1: Table S1.** Demographic characteristics of the study subjects.

**Additional file 2: Table S2.** Selected 22 significant SNPs (*P* < 10^− 4^) from the discovery cohort in 197 hypospadias cases and 933 controls.

**Additional file 3: Table S3.** Results of association between hypospadias risk and the 19 SNPs selected for replication.

**Additional file 4: Table S4.** Association of previous reported SNPs identified in European GWAS in a Chinese population.

**Additional file 5: Table S5.** Association of SNP rs11170516 with moderate hypospadias and severe hypospadias.

**Additional file 6: Table S6.** A list of SNPs with r^2^ ≥ 0.8 for risk SNP rs11170516 using HaploReg version 2 [20].

**Additional file 7: Figure S1.** The principal component analysis was performed using the first three principal components.

**Additional file 8: Figure S2.** *SP1* differentially expressed in normal tissues based on NCBI annotation.

## Data Availability

The datasets analyzed during the current study are available from the corresponding author on reasonable request.

## Abbreviations

CI: Confidence interval
cis-eQTL: Cis-expression quantitative trait locus
GTEx: Genotype-Tissue Expression
GWAS: Genome-wide association study
HWE: Hardy-Weinberg Equilibrium
LD: Linkage disequilibrium
MAF: Minor allele frequency
OR: Odds ratio
PCA: Principal component analysis
PPI: Protein-protein interaction
QC: Quality control
RAF: Risk allele frequencies
