# Supplementary material for: Genome-wide association study in Chinese cohort identifies one novel hypospadias risk associated locus at 12q13.13
Source: BMC Med Genomics. 2019 Dec 19;12:196. doi: 10.1186/s12920-019-0642-0 (PMC6923877; doi:10.1186/s12920-019-0642-0)
Supplement: Supplementary file 1 — Additional file 1: Table S1. Demographic characteristics of the study subjects. [file 12920_2019_642_MOESM1_ESM.docx]

**Table S1.** Demographic characteristics of the study subjects.

| Characteristics | Discovery stage | |  | Replication 1 | | | Replication 2 | | | |  |
| --- | --- | --- | --- | --- | --- | --- | --- | --- | --- | --- | --- |
|  | Cases (N=200) | Controls (N=1,008) |  | Cases (N=118) | | Controls (N=383) | Cases (N=137) | | Controls (N=190) | |  |
| Age (year)^a^ |  |  |  | |  | | |  | |  | |
| Mean (SD) | 2.6(2.0) | 62.1(10.0) | 3.6(3.0) | | 6.8(3.0) | | | 3.6(3.0) | | 3.4(2.6) | |
| Severity^b^ |  |  |  | |  | | |  | |  | |
| Mild | 0 | 0 | 0 | | 0 | | | 0 | | 0 | |
| Moderate | 54(27%) | 0 | 52(44%) | | 0 | | | 53(39%) | | 0 | |
| Severe | 146(73%) | 0 | 66(56%) | | 0 | | | 84(61%) | | 0 | |

^a^Age at diagnosis for cases or at recruitment for controls;

^b^The severity of hypospadias cases were classified into mild (glandular), moderate (penile), or severe (in the scrotum or perineum) according to the abnormal location of the urethral opening.
