# Supplementary material for: Genome-wide association study in Chinese cohort identifies one novel hypospadias risk associated locus at 12q13.13
Source: BMC Med Genomics. 2019 Dec 19;12:196. doi: 10.1186/s12920-019-0642-0 (PMC6923877; doi:10.1186/s12920-019-0642-0)
Supplement: Supplementary file 2 — Additional file 2: Table S2. Selected 22 significant SNPs (P < 10− 4) from the discovery cohort in 197 hypospadias cases and 933 controls. [file 12920_2019_642_MOESM2_ESM.docx]

**Table S2.**Selected 22 significant SNPs (*P*<10^-4^) from the discovery cohort in 197 hypospadias cases and 933 controls.

| CHR^a^ | SNP | Position^b^ | Risk/nonrisk allele | *P* value^c^ |
| --- | --- | --- | --- | --- |
| 1 | rs6685335 | 224881989 | C/A | 1.60×10^-8^ |
| 1 | rs7521411 | 26941436 | T/C | 1.45×10^-5^ |
| 3 | rs9812289 | 77073964 | A/G | 2.58×10^-5^ |
| 3 | rs6551343 | 88604818 | C/T | 6.77×10^-5^ |
| 4 | rs17698720 | 28549589 | G/A | 4.57×10^-6^ |
| 4 | rs34709644 | 40643872 | CA/C | 5.12×10^-6^ |
| 4 | rs2126854 | 76453149 | G/A | 7.42×10^-5^ |
| 4 | rs73842716 | 120690314 | G/T | 4.89×10^-5^ |
| 6 | rs1223552 | 13173833 | A/G | 7.71×10^-6^ |
| 7 | rs7805909 | 16382521 | C/T | 1.78×10^-5^ |
| 8 | rs76710054 | 8995131 | A/G | 3.25×10^-5^ |
| 8 | rs12674538 | 55486408 | A/G | 9.06×10^-6^ |
| 10 | rs2357221 | 17604430 | G/A | 3.07×10^-5^ |
| 12 | rs11170516 | 53752692 | G/A | 1.60×10^-6^ |
| 12 | rs7309381 | 70279419 | G/A | 3.43×10^-5^ |
| 13 | rs2057412 | 49982570 | C/T | 7.93×10^-10^ |
| 14 | rs12897826 | 41494727 | G/A | 5.85×10^-5^ |
| 15 | rs2622758 | 35548809 | A/G | 1.43×10^-5^ |
| 15 | rs11630220 | 58808174 | A/G | 2.61×10^-5^ |
| 17 | rs76843554 | 38946965 | T/C | 4.78×10^-5^ |
| 21 | rs2246042 | 31098182 | A/G | 8.64×10^-6^ |
| 23 | rs5907561 | 143472667 | T/C | 4.05×10^-5^ |

^a^Chromosome.

^b^According to GRCh37/hg19.

^c^*P* values in additive model were estimated using a logistic regression model.
