# Supplementary material for: Genome-wide association study in Chinese cohort identifies one novel hypospadias risk associated locus at 12q13.13
Source: BMC Med Genomics. 2019 Dec 19;12:196. doi: 10.1186/s12920-019-0642-0 (PMC6923877; doi:10.1186/s12920-019-0642-0)
Supplement: Supplementary file 3 — Additional file 3: Table S3. Results of association between hypospadias risk and the 19 SNPs selected for replication. [file 12920_2019_642_MOESM3_ESM.docx]

**Table S3.**Results of association between hypospadias risk and the 19 SNPs selected for replication.

|  |  |  |  | Replication 1 | | Replication 2 | |
| --- | --- | --- | --- | --- | --- | --- | --- |
| CHR^a^ | SNP | Position^b^ | Risk/nonrisk Allele^c^ | OR^c^ | *P* value^c^ | OR^c^ | *P* value^c^ |
| 1 | rs7521411 | 26941436 | T/C | 0.7(0.36-1.35) | 0.28 | 1.28(0.64-2.57) | 0.48 |
| 3 | rs9812289 | 77073964 | A/G | 0.86(0.56-1.34) | 0.51 | 1.56(0.96-2.54) | 0.08 |
| 3 | rs6551343 | 88604818 | C/T | 0.84(0.58-1.21) | 0.35 | 1.07(0.73-1.56) | 0.73 |
| 4 | rs17698720 | 28549589 | G/A | 1.05(0.69-1.6) | 0.81 | 1.04(0.68-1.61) | 0.85 |
| 4 | rs2126854 | 76453149 | T/C | 1.01(0.7-1.45) | 0.97 | 1.33(0.87-2.01) | 0.19 |
| 4 | rs73842716 | 120690314 | G/T | 0.86(0.23-3.12) | 0.81 | 0.92(0.25-3.32) | 0.9 |
| 6 | rs1223552 | 13173833 | A/G | 0.93(0.69-1.25) | 0.62 | 1.18(0.83-1.67) | 0.36 |
| 8 | rs76710054 | 8995131 | A/G | 0.77(0.38-1.55) | 0.46 | 1.33(0.63-2.79) | 0.45 |
| 8 | rs12674538 | 55486408 | A/G | 1.05(0.78-1.42) | 0.73 | 1.18(0.87-1.6) | 0.29 |
| 10 | rs2357221 | 17604430 | G/A | 0.77(0.56-1.05) | 0.1 | 1.14(0.83-1.55) | 0.41 |
| 12 | rs11170516 | 53752692 | G/A | 0.59(0.38-0.88) | 1.3×10^-2^ | 0.56(0.38-0.86) | 8.0×10^-3^ |
| 12 | rs7309381 | 70279419 | G/A | 0.91(0.59-1.42) | 0.69 | 0.94(0.57-1.56) | 0.82 |
| 13 | rs2057412 | 49982570 | C/T | 1.07(0.73-1.58) | 0.73 | 1.22(0.81-1.83) | 0.35 |
| 14 | rs12897826 | 41494727 | G/A | 1.09(0.79-1.5) | 0.6 | 1.06(0.74-1.5) | 0.76 |
| 15 | rs2622758 | 35548809 | A/G | 0.92(0.68-1.25) | 0.6 | 1.1(0.77-1.57) | 0.58 |
| 15 | rs11630220 | 58808174 | A/G | 0.89(0.42-1.88) | 0.76 | 0.7(0.36-1.36) | 0.29 |
| 17 | rs76843554 | 38946965 | T/C | 0.91(0.44-1.91) | 0.81 | 2.31(1.08-4.99) | 0.03 |
| 21 | rs2246042 | 31098182 | A/G | 1.29(0.88-1.9) | 0.19 | 1.06(0.7-1.6) | 0.78 |
| X | rs5907561 | 143472667 | T/C | 0.99(0.8-1.23) | 0.95 | NA | NA |

^a^Chromosome.

^b^According to GRCh37/hg19.

^c^ORs, 95% CIs and corresponding *P* values in additive model were estimated using a logistic regression model.
