# Supplementary material for: Genome-wide association study in Chinese cohort identifies one novel hypospadias risk associated locus at 12q13.13
Source: BMC Med Genomics. 2019 Dec 19;12:196. doi: 10.1186/s12920-019-0642-0 (PMC6923877; doi:10.1186/s12920-019-0642-0)
Supplement: Supplementary file 4 — Additional file 4: Table S4. Association of previous reported SNPs identified in European GWAS in a Chinese population. [file 12920_2019_642_MOESM4_ESM.docx]

**Table S4.**Association of previous reported SNPs identified in European GWAS in a Chinese population.

| CHR^a^ | SNP | Position^b^ | Risk/nonrisk allele | *P* value^c^ |
| --- | --- | --- | --- | --- |
| 2 | rs988958 | 42287306 | A/G | 0.27 |
| 2 | rs3816183 | 43015719 | A/G | 0.40 |
| 2 | rs1918690 | 84906915 | A/G | 0.53 |
| 3 | rs2999052 | 127892037 | G/A | 0.55 |
| 4 | rs13124827 | 157340210 | A/G | 0.59 |
| 6 | rs417096 | 39727833 | A/G | 0.53 |
| 7 | rs1801085 | 27168590 | G/A | 0.76 |
| 7 | rs10214930 | 27784949 | A/G | 0.44 |
| 8 | rs16937456 | 72077955 | G/A | 0.73 |
| 8 | rs17262815 | 130491165 | C/T | 0.88 |
| 10 | rs10762738 | 78695467 | G/A | 0.94 |
| 15 | rs3743104 | 33023985 | A/G | 0.57 |
| 16 | rs17208368 | 55072007 | A/C | 0.66 |
| 16 | rs6499755 | 55341135 | G/A | 0.39 |
| 16 | rs1858800 | 73024276 | A/G | 0.43 |
| X | rs4554617 | 50203402 | C/A | 1.4×10^-3^ |

^a^Chromosome.

^b^According to GRCh37/hg19.

^c^*P* values in additive model were estimated using a logistic regression model.
