# Supplementary material for: Genome-wide association study in Chinese cohort identifies one novel hypospadias risk associated locus at 12q13.13
Source: BMC Med Genomics. 2019 Dec 19;12:196. doi: 10.1186/s12920-019-0642-0 (PMC6923877; doi:10.1186/s12920-019-0642-0)
Supplement: Supplementary file 5 — Additional file 5: Table S5. Association of SNP rs11170516 with moderate hypospadias and severe hypospadias. [file 12920_2019_642_MOESM5_ESM.docx]

**Table S5.** Association of SNP rs11170516 with moderate hypospadias and severe hypospadias

| SNP | Risk Allele | Location | Attributed genes | Classification of hypospadias | RAF^a^ | | OR(95%CI)^b^ | *P* value^b^ |
| --- | --- | --- | --- | --- | --- | --- | --- | --- |
|  |  |  |  |  | Cases | Controls |  |  |
| rs11170516 | G | 12:53752692 | *SP1*, *SP7* | Moderate | 0.869 | 0.784 | 1.83(1.32-2.60) | 4.9×10^-4^ |
|  |  |  |  | Severe | 0.881 | 0.784 | 2.03(1.57-2.66) | 1.6×10^-7^ |

^a^Risk allele frequency (RAF).

^b^ORs, 95% CIs and corresponding *P* values in additive model were estimated using a logistic regression model.
