# Supplementary material for: Genome-wide association study in Chinese cohort identifies one novel hypospadias risk associated locus at 12q13.13
Source: BMC Med Genomics. 2019 Dec 19;12:196. doi: 10.1186/s12920-019-0642-0 (PMC6923877; doi:10.1186/s12920-019-0642-0)
Supplement: Supplementary file 6 — Additional file 6: Table S6. A list of SNPs with r2 ≥ 0.8 for risk SNP rs11170516 using HaploReg version 2 [20]. [file 12920_2019_642_MOESM6_ESM.docx]

**Table S6.**A list of SNPs with r^2^ ≥ 0.8 for risk SNP rs11170516 using HaploReg version 2[[20](#_ENREF_20)].

| CHR^a^ | Position^b^ | LD (r²) | LD (D') | SNP | Ref | Alt | GENCODE genes | func annot |
| --- | --- | --- | --- | --- | --- | --- | --- | --- |
| 12 | 53774779 | 0.89 | 0.96 | rs36065378 | A | G | *SP1* | intronic |
| 12 | 53775742 | 0.81 | 0.94 | rs3832826 | CAA | C | *SP1* | intronic |
| 12 | 53777171 | 0.82 | 0.94 | rs3741651 | A | G | *SP1* | synonymous |
| 12 | 53779295 | 0.8 | 0.92 | rs71443295 | CTT | C | *SP1* | intronic |
| 12 | 53779389 | 0.8 | 0.92 | rs71443296 | A | AT | *SP1* | intronic |
| 12 | 53779775 | 0.82 | 0.94 | rs10876449 | T | C | *SP1* | intronic |
| 12 | 53782628 | 0.82 | 0.94 | rs11170525 | G | T | *SP1* | intronic |
| 12 | 53782959 | 0.82 | 0.94 | rs12368491 | G | A | *SP1* | intronic |
| 12 | 53784913 | 0.8 | 0.92 | rs7315782 | G | A | *SP1* | intronic |
| 12 | 53786033 | 0.82 | 0.94 | rs7134665 | C | T | *SP1* | intronic |
| 12 | 53787866 | 0.82 | 0.94 | rs149919208 | A | G | *SP1* | intronic |
| 12 | 53788335 | 0.82 | 0.94 | rs11170532 | G | A | *SP1* | intronic |
| 12 | 53790450 | 0.82 | 0.94 | rs7300593 | T | C | *SP1* | intronic |
| 12 | 53790985 | 0.82 | 0.94 | rs7133236 | A | G | *SP1* | intronic |
| 12 | 53791630 | 0.82 | 0.94 | rs7955418 | G | A | *SP1* | intronic |
| 12 | 53792914 | 0.82 | 0.94 | rs74090765 | T | G | *SP1* | intronic |
| 12 | 53793209 | 0.82 | 0.94 | rs35969688 | G | A | *SP1* | intronic |
| 12 | 53794787 | 0.82 | 0.94 | rs57676448 | C | T | *SP1* | intronic |
| 12 | 53798808 | 0.8 | 0.93 | rs34148420 | T | C | *SP1* | intronic |
| 12 | 53803633 | 0.8 | 0.93 | rs7968637 | T | A,C | *SP1* | intronic |
| 12 | 53804307 | 0.8 | 0.93 | rs12817984 | T | G | *SP1* | intronic |
| 12 | 53807907 | 0.8 | 0.93 | rs35937229 | CAG | C | *SP1* | 3'-UTR |

^a^Chromosome.

^b^According to GRCh37/hg19.
