# Supplementary figures and images for: Genome-wide association study in Chinese cohort identifies one novel hypospadias risk associated locus at 12q13.13
Source: BMC Med Genomics. 2019 Dec 19;12:196. doi: 10.1186/s12920-019-0642-0 (PMC6923877; doi:10.1186/s12920-019-0642-0)

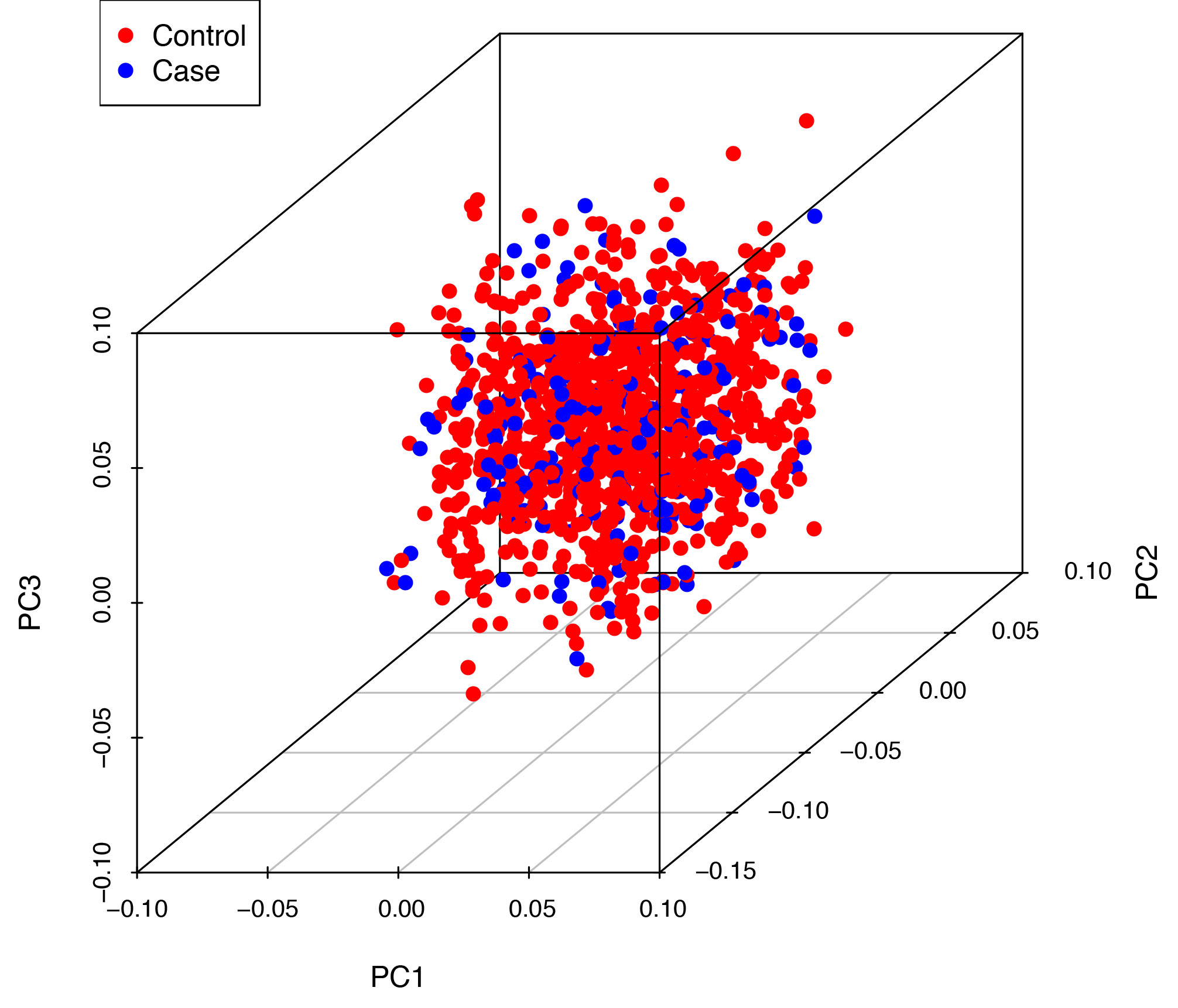

Supplement: Supplementary file 7 — Additional file 7: Figure S1. The principal component analysis was performed using the first three principal components. [file 12920_2019_642_MOESM7_ESM.jpg]

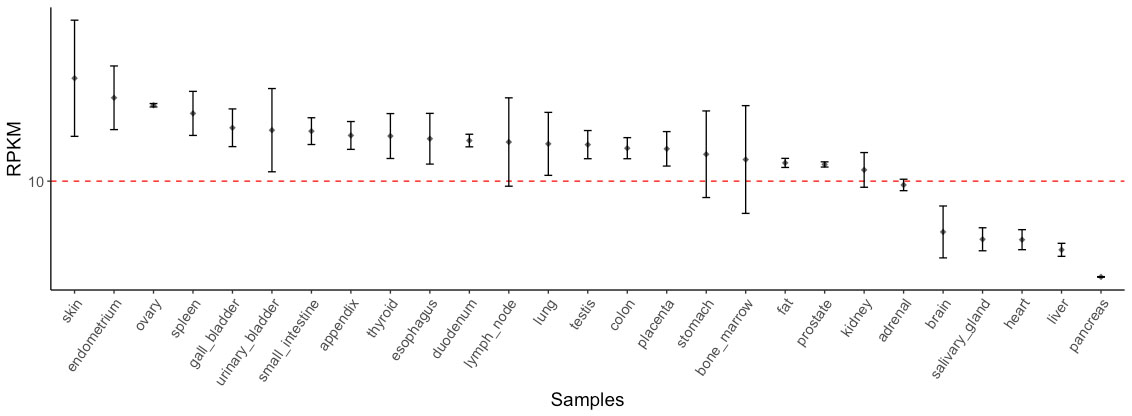

Supplement: Supplementary file 8 — Additional file 8: Figure S2. SP1 differentially expressed in normal tissues based on NCBI annotation. [file 12920_2019_642_MOESM8_ESM.jpg]
